# Spatiotemporal Variability of Dimethylsulphoniopropionate on a Fringing Coral Reef: The Role of Reefal Carbonate Chemistry and Environmental Variability

**DOI:** 10.1371/journal.pone.0064651

**Published:** 2013-05-28

**Authors:** Heidi L. Burdett, Penelope J. C. Donohue, Angela D. Hatton, Magdy A. Alwany, Nicholas A. Kamenos

**Affiliations:** 1 School of Geographical and Earth Sciences, University of Glasgow, Glasgow, United Kingdom; 2 Scottish Association for Marine Science, Oban, Argyll, United Kingdom; 3 Department of Marine Science, Suez Canal University, Ismailia, Egypt; University of New South Wales, Australia

## Abstract

Oceanic pH is projected to decrease by up to 0.5 units by 2100 (a process known as ocean acidification, OA), reducing the calcium carbonate saturation state of the oceans. The coastal ocean is expected to experience periods of even lower carbonate saturation state because of the inherent natural variability of coastal habitats. Thus, in order to accurately project the impact of OA on the coastal ocean, we must first understand its natural variability. The production of dimethylsulphoniopropionate (DMSP) by marine algae and the release of DMSP’s breakdown product dimethylsulphide (DMS) are often related to environmental stress. This study investigated the spatiotemporal response of tropical macroalgae (*Padina* sp., *Amphiroa* sp. and *Turbinaria* sp.) and the overlying water column to natural changes in reefal carbonate chemistry. We compared macroalgal intracellular DMSP and water column DMSP+DMS concentrations between the environmentally stable reef crest and environmentally variable reef flat of the fringing Suleman Reef, Egypt, over 45-hour sampling periods. Similar diel patterns were observed throughout: maximum intracellular DMSP and water column DMS/P concentrations were observed at night, coinciding with the time of lowest carbonate saturation state. Spatially, water column DMS/P concentrations were highest over areas dominated by seagrass and macroalgae (dissolved DMS/P) and phytoplankton (particulate DMS/P) rather than corals. This research suggests that macroalgae may use DMSP to maintain metabolic function during periods of low carbonate saturation state. In the reef system, seagrass and macroalgae may be more important benthic producers of dissolved DMS/P than corals. An increase in DMS/P concentrations during periods of low carbonate saturation state may become ecologically important in the future under an OA regime, impacting larval settlement and increasing atmospheric emissions of DMS.

## Introduction

The carbonate chemistry of the oceans is regulated by a carbonate equilibrium that is driven by the dissolution of atmospheric CO_2_ into the oceans. Since the Industrial Revolution, atmospheric CO_2_ concentrations have increased from 280 ppm to ∼390 ppm; CO_2_ concentrations have been increasing by ∼2 ppm yr^−1^ since 2000 [1]. It is projected that continued anthropogenic emissions of CO_2_ will cause the pH of the oceans to drop by 0.3–0.5 units by 2100 [2], a process known as ocean acidification (OA). This rate of change has been estimated to be ∼100 times faster than during glacial terminations [3], raising concerns over the future survival of calcifying organisms.

Coastal habitats such as coral reefs are characterised by more extreme natural variations in carbonate saturation state compared to the open ocean, reflecting diurnal and seasonal cycles driven by biological and physical processes [3]. Thus, in order to accurately project the impact of an OA scenario on coastal ecosystems, one must first understand contemporary natural variability. Spatial heterogeneity of carbonate chemistry has been observed in coral reef systems, with reef flats and lagoons more variable than fringing forereefs due to biological (benthos composition) and physical (wave action and residence time) variables [4], [5], [6], [7], [8]. Similarly, surface *p*CO_2_ in atoll and barrier reef lagoons may be higher than offshore waters [9]. Where the benthos is dominated by filamentous turf algae, pH may be high (i.e. less acidic) [7]. Short-term and longer-term temporal heterogeneity in reefal carbonate chemistry has also been observed. During the day, net calcification may be observed, whilst net dissolution may be observed at night [8], influencing the carbonate chemistry of the overlying water column. Seasonal patterns in carbonate chemistry have been observed [10] but this may be de-coupled from reef calcification due to the maintenance of threshold saturation levels during the winter [11].

The response of calcified organisms to reduced carbonate saturation state is varied depending on their carbonate structure, their method of calcification and their ability to benefit from an increase in CO_2_ (e.g. for photosynthesis) [12]. This has led to the suggestion that, under an OA scenario, coral reefs may become dominated by macroalgae (fleshy and coralline) [13]. The negative effect of low carbonate saturation state on the calcification and growth of red coralline algae (which deposit high-magnesium calcite, the most thermodynamically unstable carbonate polymorph) has been well reported. During the winter, net calcification in the temperate/polar coralline alga *Lithothamnion glaciale* was significantly reduced at high CO_2_ (750, 950 and 1500 ppm), whilst only the very high CO_2_ treatment (1500 ppm) induced a decrease in calcification in the summer [14]. Growth rates and structural integrity of *L. glaciale* may also be reduced under high CO_2_ (589–1080 µatm) [15], [16]. After a 1 year exposure to high CO_2_ conditions (700 ppm), net dissolution exceeded net calcification in *Lithophyllum cabiochae* [17], and when combined with high temperature (+3°C) more algal necroses, death and dissolution were observed [17]. Spore production, growth and recruitment of the Corallinaceae are also inhibited by high CO_2_ conditions (550–760 ppm) [18], [19], [20]. Additionally, high CO_2_ (1000 ppm) and UV radiation (particularly UVB) may act synergistically to inhibit growth, photosynthetic O_2_ evolution and calcification in the geniculate coralline alga *Corallina sessilis* [21].

In the field, calcifying organisms may decrease in abundance under high, variable *p*CO_2_ conditions [22]. Despite this, *Padina* spp. (Ochrophyta: Dictyoaceae), one of only two known calcifying brown algae, appear to thrive in CO_2_ vent systems, albeit with reduced calcification in the more acidified areas [23], attributed to the lower abundance of grazing sea urchins in acidified areas and enhanced photosynthesis from higher CO_2_ availability [23]. Experimental reef studies (conducted in 2650 m^3^ mesocosms dominated by macroalgae) suggest that although calcification appears to decline under high CO_2_ conditions, net organic production does not change [24], [25]. However, current studies are not wholly conclusive and more detailed investigations into the biochemical and morphological effects of low carbonate saturation state on calcifying benthic macroalgae are still required.

Dimethylsulphoniopropionate (DMSP) is a sulphur compound produced by many marine algae and is the major precursor to dimethylsulphide (DMS), a gas that may be linked to local climate regulation through aerosol production and cloud formation [26], [27]. A number of cellular functions have been described for DMSP in macro- and microalgae, including as a compatible solute [28], a cryoprotectant [29], an antioxidant [30] and a herbivore deterrent [31] and attractant [32]. A general reduction in intracellular DMSP concentrations with increasing latitude has been suggested for macroalgae in the northern hemisphere [31], perhaps in response to the cryoprotective properties of DMSP [29]. However, this suggestion was based primarily on Chlorophyta species, the abundance of which also increases with increasing latitude. In contrast, other macroalgal secondary metabolites (e.g. terpenes) tend to increase in low latitudes, perhaps due to increased grazing pressure [33]. The principal functions of DMSP have not been extensively studied and the lack of data available on macroalgal DMSP concentrations currently prevents such assessments to be made.

Information on the effect of reduced carbonate saturation state on intracellular DMSP concentrations in algae is limited and has provided variable results. Intracellular DMSP concentrations in the green macroalgae *Ulva lactuca* and *U. clathrata* was not affected by elevated *p*CO_2_ conditions (up to 1514 µatm) [34]. However, in the non-geniculate red coralline alga *L. glaciale*, reduced pH (pH 7.7), particularly when variable, led to an increase in intracellular DMSP concentrations [16]. Species-specific responses have also been observed in phytoplankton. An up-regulation of intracellular DMSP has been observed in *Emiliania huxleyi* under high temperature and high CO_2_ (+4°C/1000 ppm) [35]; (+6°C/790 ppm) [36]. In contrast, intracellular DMSP concentrations were reduced in *Thalassiosira pseudonana* and *Phaeodactylum tricornutum* under high temperature and CO_2_ (+6°C/790 ppm) [36].

In tropical reef environments, intracellular DMSP may be important as an antioxidant, grazing deterrent and/or compatible solute. Recent studies also suggest that intracellular DMSP may play a role in improving tolerance to variable carbonate chemistry conditions [16]. Corals from environmentally variable conditions (e.g. reef lagoons and flats) may be more thermally tolerant than those from more stable conditions (e.g. reef crests) [37]. Thus, it may be expected that organisms (e.g. corals and macroalgae) on reef flats have also developed mechanisms (e.g. up-regulation of DMSP) to become more tolerant of carbonate system variability compared to those on reef crests. This study assessed natural spatiotemporal variability of intracellular DMSP and water column DMSP+DMS (DMS/P) concentrations in the fringing reef environment of the Red Sea and related this to the carbonate chemistry of the overlying water column. It was hypothesised that (1) intracellular DMSP and water column DMS/P concentrations would fluctuate more on the environmentally variable reef flat compared to the environmentally stable reef crest and (2) corals would be the primary source of DMS/P in the reef system, as was observed on the Great Barrier Reef, Australia [38], [39].

## Materials and Methods

### Study Site and Field Sampling Design

Research was conducted on the fringing Suleman Reef, Gulf of Aqaba, northern Red Sea, Egypt (28°28′N, 34°30′W) in August 2011. This reef is characterised by four distinct zones: seagrass beds nearest the shore, the reef flat 40–60 m from the shore, the reef crest 100–120 m from the shore and the reef slope which extends away from the reef crest to a depth of ∼10 m. On average, water depth was uniform across the reef platform (∼0.8 m). All research was approved by the Dahab Marine Research Center and the Marine Environmental Center, Suez Canal University, provided the permit to conduct the research.

Water and macroalgal samples were taken at the reef flat (dominated by fleshy and coralline macroalgae) and the reef crest (dominated by small branching corals, macroalgae and encrusting coralline algae) over two 45-hour experimental periods, at T14, T21, T29, T38 and T45 hours (Where T0 was 00∶00 on day one). *In situ* water temperature, salinity and dissolved oxygen were recorded at each timepoint using a YSI Pro 2030 instrument. *In situ* photosynthetically active radiation (PAR) at the sea bed was recorded hourly at the reef flat and reef crest using an Apogee QSO-E underwater quantum sensor and Gemini voltage data logger.

### Carbonate Chemistry

At each timepoint, water samples were preserved for carbonate chemistry with MgCl_2_ [40] and stored in the dark for subsequent total alkalinity (TA) and dissolved inorganic carbon (DIC) analysis. DIC was determined using a CO_2_ Coulometer (CM5014 v.3, UIC Ltd.) with acidification module (CM5130 v.2, UIC Ltd.) and methods described by Dickson et al. [40]. TA was determined using the 2-stage open-cell titration method [40]. *In situ* TA, DIC, temperature and salinity measurements were used to calculate pH, *p*CO_2_, HCO_3_^−^ and CO_3_^2−^ concentrations and calcite (ΩCa) and aragonite (ΩAr) saturation states using CO2SYS [41] with dissociation constants from Mehrbach et al. [42] refit by Dickson and Millero [43] and [KSO_4_] using Dickson [44].

### Intracellular DMSP

Three macroalgal species were analysed for intracellular DMSP (n = 10 per species, per timepoint, per location): *Padina* sp. (Ochrophyta: Dictyotales), *Amphiroa* sp. (Rhodophyta: Corallinales) and *Turbinaria* sp. (Ochrophyta: Fucales). Both *Padina* sp. and *Amphiroa* sp. were found on the reef crest and reef flat; *Turbinaria* sp. was found only on the reef crest. Macroalgal samples (∼0.2 g) were stored in 10 M NaOH in gas-tight glass vials sealed with Pharma-Fix (Grace Alltech) crimp lids to hydrolyse all intracellular DMSP into DMS. Vials were stored in the dark until DMS quantification.

### Water Column DMSP

At each sampling timepoint, water samples were collected from the reef flat and reef crest (n = 5 per location per timepoint), from 15 cm above the seabed. 50 ml water samples were filtered using a 0.7 µm depth filter (Millepore). The filtrate and filter paper were stored in glass vials sealed with Pharma-Fix septa at a final concentration of 0.33 M NaOH. These samples represented the total dissolved and particulate DMS+DMSP pool (DMSPt). Logistical constraints limited DMSPt sample collections to the first 45-hour sampling period only.

### Reef Transect – Spatial Variation

In addition to the reef flat/reef crest diel comparison, water samples (n = 3 per location) were also taken at one timepoint (16∶00) along a transect perpendicular to the shore at five locations: at the seagrass bed (10 m from the shore), on the reef flat (50 m from the shore), crest (100 m from the shore) and slope (120 m from the shore) and 270 m offshore (sandy bottom, no discernible macro-primary production). Samples were prepared for DMSPt using the methods described above.

### DMSP Analysis

All DMSP samples were analysed using a Shimadzu 2014 gas chromatograph (GC) fitted with a 25 m capillary column (Restek RTx-5MS 30 m column, 0.25 mm ID) and a sulphur-specific FPD detector (injector port and column oven temperature: 45°C, detector: 200°C). Samples were analysed within 2 months of collection. Intracellular DMSP samples were analysed by direct injection of the vial headspace into the GC injector port. Water column DMSPt samples were pre-concentrated using the purge and cryotrap technique [45] before injection into the GC. Sample concentrations were quantified from DMSP standard calibration curves (DMSP standard from Research Plus Inc.). The limit of detection was 960 ng S per 100 µl headspace injection and 0.64 ng S per injection using the cryotrap system; standard and sample precision was within 3%.

### Proportion Calcified

The proportion of *Padina* sp. and *Amphiroa* sp. from the reef crest and flat that was calcified was determined by recording the mass of samples before and after overnight storage in 10 M NaOH to break down the organic material. The mass remaining represented the calcified portion, which was expressed as a percentage of the original mass. The proportion of *Amphiroa* sp. from the reef crest that was calcified could not be calculated as the carbonate structure broke down in the NaOH preventing final mass assessment.

### Statistics

Intracellular DMSP measurements could not be transformed to achieve normality and homogeneity of variance, thus a non-parametric multi-comparison Kruskall-Wallis test was performed to compare the data sets. Transect DMSPt data were analysed using general linear models (test assumptions met). Analyses were conducted using Minitab V15.

Generalised Additive Models (GAMs) were used to assess the relationship between the reefal abiotic parameters and measured macroalgal and water column DMSP concentrations. GAMs were conducted in R (V2.14.2) using the ‘mgcv’ package [46]. Thin plate regression splines were used as the smoothing basis, allowing multiple predictors to be considered and reducing subjectivity as knot locations (i.e. where splines join) are not manually specified [46]. Abiotic parameter interactions were identified using the ‘tree’ package in R. The most parsimonious GAM for each DMSP dataset was determined using generalised cross validation techniques (GCV) [46].

## Results

### Water Chemistry

**Figure 1:**
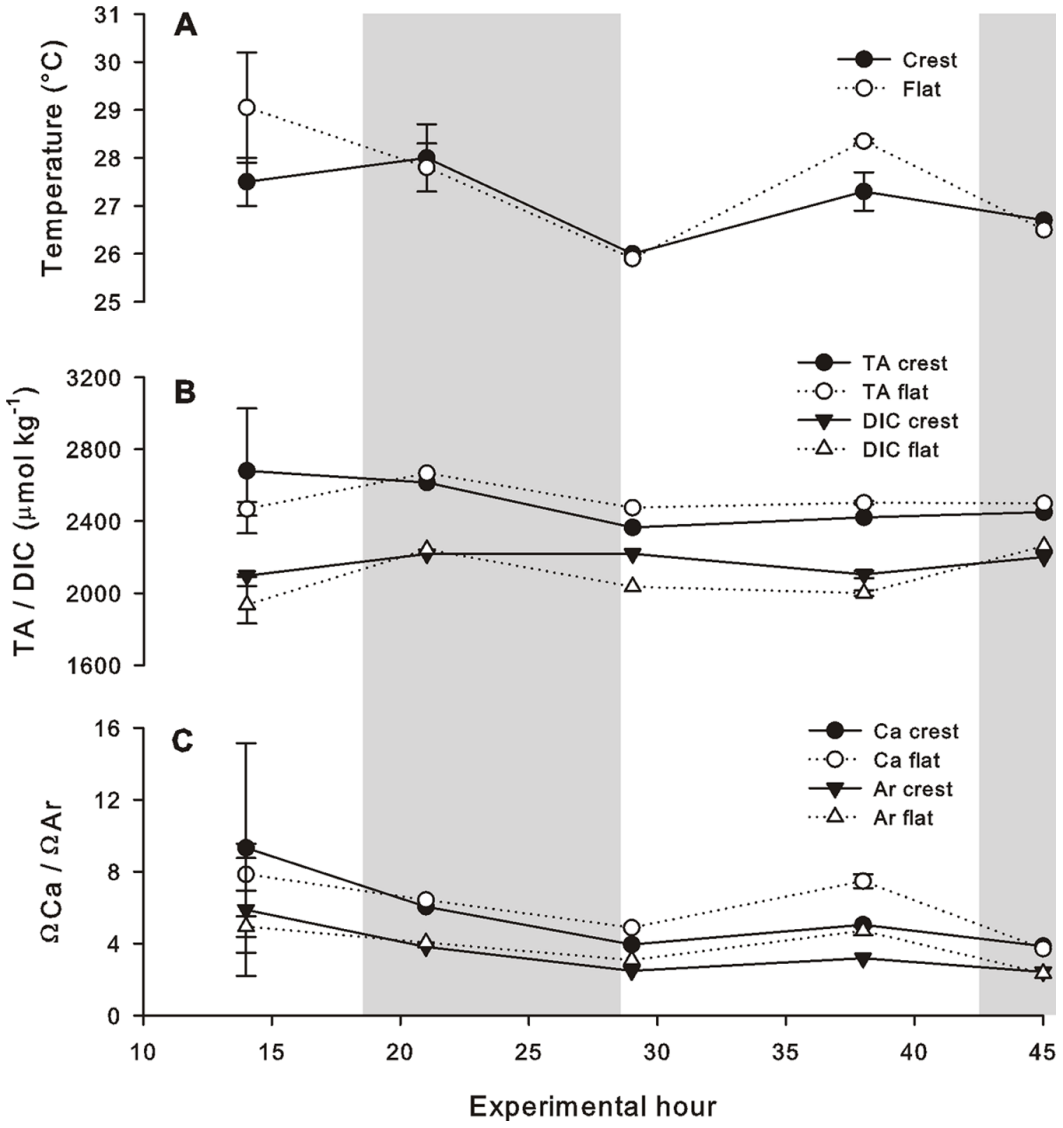
Diel pattern of abiotic parameters on Suleman Reef, Egypt. *In situ* A: water temperature (°C), B: TA and DIC (µmol kg^−1^) and C: calcite (ΩCa) and aragonite (ΩAr) saturation states at the reef crest (black symbols) and reef flat (open symbols) over a 45-hour period on Suleman Reef, Egypt. Grey shading indicates night-time (sunset – sunrise). Data presented as mean±0.5 range of two experimental runs.

Both the reef crest and reef flat exhibited a diel trend in water temperature: highest water temperatures were in the afternoon and lowest temperatures were at dawn (Figure 1A). Whilst minimum water temperature on the reef flat and reef crest were the same (26.0°C), maximum temperatures were higher on the reef flat than the reef crest (29.1°C compared to 27.5°C), resulting in the reef flat having a wider temperate range than the reef crest (3.15°C and 1.5°C respectively, Figure 1A). The range in dissolved oxygen was also wider on the reef flat (60–160%) than the reef crest (110–70%, Figure S1A, supporting information). No difference was observed between the reef crest and reef flat in terms of PAR (0.8 m depth, Figure S1B, supporting information), with a maximum of >1500 µmol photons m^−2^ s^−1^ at 14∶00 on day one and day two of the 45-hour experimental periods.

TA did not exhibit a strong diurnal oscillation on the reef crest (2422–2681 µmol kg^−1^) or flat (2433–2574 µmol kg^−1^, Figure 1B). DIC on the reef flat (<2000 µmol kg ^−1^) was lower than the reef crest (∼2100 µmol kg^−1^) at all daytime sampling points; at night (21 h and 45 h) DIC on the flat and crest were similar (2100–2200 µmol kg^−1^, Figure 1B). Saturation states were highest in the day (maximum ΩCa: 9.3 on the crest and 7.8 on the flat; ΩAr: 5.9 on the crest and 4.9 on the flat) and lowest at night/dawn (minimum ΩCa: 3.9 on the crest and 3.7 on the flat; ΩAr: 2.4 on the crest and 2.3 on the flat, Figure 1C). pH, CO_3_^2−^ and HCO_3_^−^ concentrations and *p*CO_2_ diel trends (derived from TA and DIC) are available in Figure S1C–F (supporting information).

### Intracellular and Water Column DMSP

**Figure 2:**
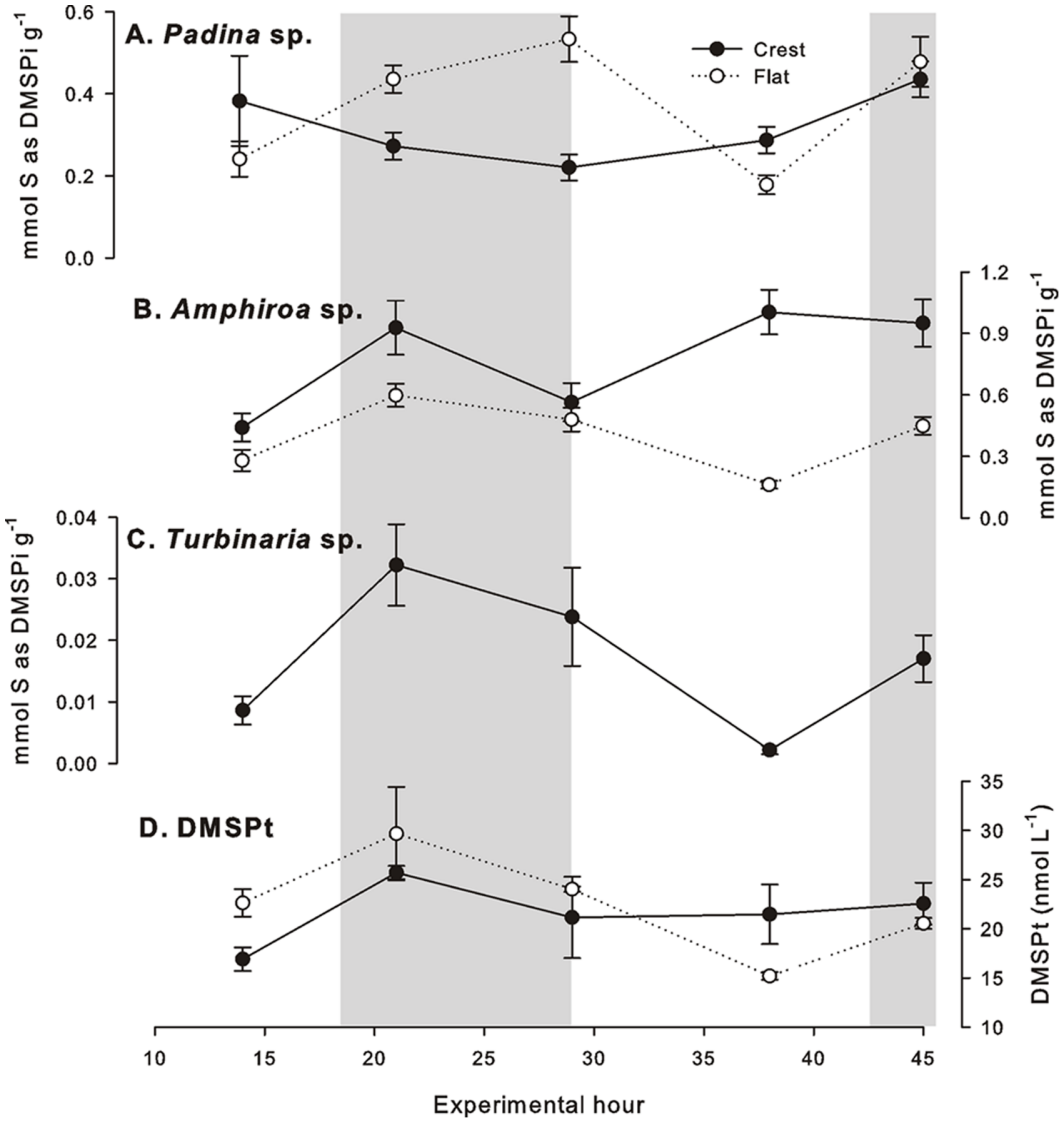
Diel pattern of DMS/P measurements on Suleman Reef, Egypt. Intracellular DMSP (mg S g^−1^ algae as intracellular DMSP, DMSPi) of A: *Padina* sp., B: *Amphiroa* sp. and C: *Turbinaria* sp. and, in the water column, D: total DMS+DMSP (DMSPt, nmol L^−1^) on the reef crest (black circles) and reef flat (open circles) over a 45-hour period on Suleman Reef, Egypt. Grey shading indicates night-time (sunset – sunrise). Data presented as mean±SE. Note the different y-axes on all graphs.

A general diurnal oscillation was observed for most DMSP measurements: high DMSP concentrations at 21∶00 (T21+T45) and low concentrations at 14∶00 (T14+T38, Figure 2). An exception to this was *Padina* sp. on the crest (no diurnal trend, Figure 2A). No statistical difference was observed between the intracellular DMSP concentrations of *Padina* sp. from the reef flat and crest except at T29 (Z = 3.41, p<0.001, Figure 2A). Intracellular DMSP in *Amphiroa* sp. on the flat (12.6±5.5 mg S g^−1^_,_ mean±SD) was significantly lower than the crest (24.9±8.2 mg S g^−1^, Z = 4.73, p<0.001, Figure 2B), with a significant diurnal trend of low intracellular DMSP at 14∶00 (T14+T38) and high DMSPi at 21∶00 (T21+T45, Z = 4.63, p<0.001, Figure 2B). A diurnal trend was also evident in the intracellular DMSP concentrations of *Turbinaria* sp. from the reef crest (<0.3 mg S g^−1^ at 14∶00, >0.5 mg S g^−1^ at 21∶00 [T21+T45]), although this was not significant (Z <3.40) and was characterised by a relatively high variability at 21∶00 (T21+T45) and 07∶00 (T29, Figure 2C). DMSPt did not differ significantly between the crest and flat (T_22_ = −0.18, p = 0.862, Figure 2D), but there was suggestion of a diurnal trend, with elevated concentrations at 21∶00 (T21+T45) compared to 14∶00 (T14+T38), particularly on the reef flat.

### Associations between Environmental Conditions and DMSP

Carbonate chemistry parameters were present in the most parsimonious GAM models for all DMSP datasets (Table 1). Water temperature was significantly associated with intracellular DMSP concentrations in *Amphiroa* sp. (reef crest and flat, Table 1). Dissolved oxygen was significantly associated with intracellular DMSP concentrations in *Padina* sp. (reef crest), *Turbinaria* sp. (reef crest) and water column DMSPt (reef flat, Table 1). The % deviance explained by the GAM models was generally higher for reef flat datasets, except *Padina* sp. (reef crest: 66%, reef crest: 44% deviance explained, Table 1).

**Table 1:** Most parsimonious GAM models for intracellular DMSP (DMSPi) for *Padina* sp., *Amphiroa* sp. and *Turbinaria* sp. and water column DMSP on the reef crest and flat at Suleman Reef, Egypt.

Location	Sample	GAM formula	Est. df	GCV score	Adj. R^2^	Dev. exp. (%)
Crest	*Padina*	DMSPi ∼ s(TA)+s(DO)	6.92	0.084	0.64	66.4
	*Amphiroa*	DMSPi ∼ s(TA)+s(Temp:TA)	5.16	0.074	0.278	31.3
	*Turbinaria*	DMSPi ∼ s(DO)+s(DO:DIC)	4.00	0.150	0.536	56.2
	Water column	DMSPt ∼ s(ΩCa)	2.57	0.009	0.169	27
Flat	*Padina*	DMSPi ∼ s(TA)+s(TA:ΩCa)	5.02	0.100	0.408	43.7
	*Amphiroa*	DMSPi ∼ s(Temp)+s(ΩCa)+s(*p*CO_2_)	5.99	0.034	0.739	75.4
	Water column	DMSPt ∼ s(DO)+s(pH)	3.98	0.005	0.745	80.9

Statistics presented: estimated degrees of freedom (Est. df), GCV score, Adjusted R^2^ (Adj. R^2^), % deviance explained (Dev. exp.).

TA: Total alkalinity, DO: dissolved oxygen, Temp: water temperature, DIC: dissolved inorganic carbon, ΩCa: calcite saturation state, *p*CO_2_: partial pressure of CO_2_.

### Transect DMSPt

**Figure 3:**
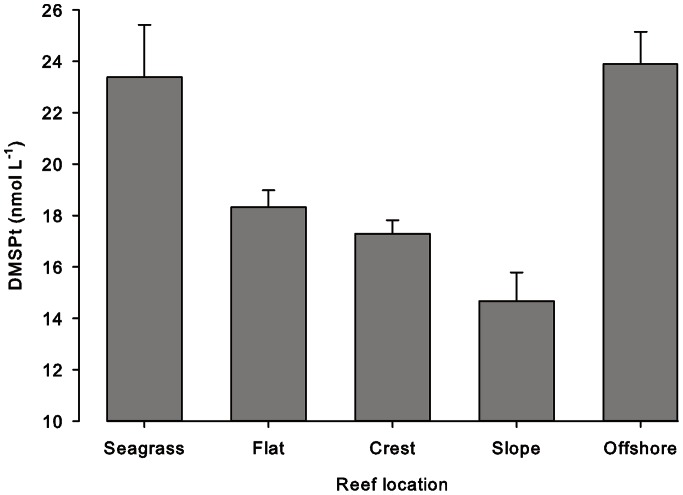
DMSPt measurements along a transect across Suleman Reef, Egypt. Measurements taken from the five zones of Suleman reef: seagrass beds nearest the shore, the reef flat, reef crest, reef slope and 270 m offshore. Data presented as mean±SE.

A significant difference in DMSPt was observed between the five transect sampling sites (F_4_ = 10.38, p = 0.001) (Figure 3). DMSPt on the reef crest (17.29±0.53 nmol L^−1^, mean±SE) and slope (14.67±1.11 nmol L^−1^) were significantly lower than the seagrass (23.38±2.04 nmol L^−1^) and offshore (23.89±1.29 nmol L^−1^) sites. The reef flat exhibited an intermediate DMSPt concentration (18.32±0.66 nmol L^−1^).

### Proportion Calcified

No significant difference in the proportion of thallus that was calcified was observed between *Padina* sp. samples from the reef crest (56.5±16.0%, mean±SD) and flat (51.5±4.7%, T_1_ = 0.30, p = 0.816). The proportion for *Amphiroa* sp. on the reef flat that was calcified was 43.6±5.0%. The proportion of *Amphiroa* sp. on the reef crest that was calcified could not be determined, although visually these samples appeared to be more heavily calcified.

## Discussion

### Biological Control of Carbonate Chemistry

Biological processes, particularly photosynthesis/respiration and calcification/dissolution can strongly influence the carbonate chemistry of tropical reefs [4], [7], [8]. The Suleman reef platform was, on average, only 0.8 m deep, thus biological processes are likely to have strongly influenced the observed diel patterns in carbonate chemistry, probably due to the low surface area to volume ratio [47]. In general, carbonate parameters were more variable on the reef flat compared to the reef crest. The diel difference between CO_2_ utilisation and release was likely higher on the reef flat than the crest due to the high proportion of macroalgal cover on the reef flat. CO_2_ uptake for photosynthesis during the day (which reduces DIC) and release by respiration at night (which increases DIC) were the probable drivers of the diel pattern in DIC concentrations. This proposed biological control mechanism was also reflected in the dissolved oxygen concentrations, which were ∼160% saturated during the day (release from photosynthesis) and <60% saturated during the night (uptake for respiration) on the reef flat (see Figure S1A, supporting information). The TA and DIC measurements observed in Suleman Reef are within the range cited for other tropical reef systems [47]. Such large diel changes in oxygen saturation have also been reported for Puerto Rican reefs (62–138%) [6].

### Species-specific Intracellular DMSP Concentrations

Intracellular DMSP concentrations of Red Sea tropical macroalgae in this study were higher than intracellular DMSP concentrations reported for the same genera from Australia [39] and the Caribbean [48]. Restricted water circulation within the Gulf of Aqaba results in more extreme conditions (salinity, temperature and carbonate chemistry) compared to other tropical reefs [49], which may necessitate an increase in intracellular DMSP relative to other tropical reef environments. Despite this, inter- and intra-species differences in intracellular DMSP concentrations were observed in the macroalgae of Suleman Reef, with *Amphiroa* sp. (crest)>*Amphiroa* sp. (flat) >*Padina* sp. (crest and flat)>*Turbinaria* sp. (crest). Grazing pressure may have contributed to the species-specific and diel patterns in intracellular DMSP concentrations, as grazing pressure is typically highest at night on tropical reefs [50] and DMSP may be an important grazing defence mechanism in macroalgae [51], [52], [53].

### Diel Regulation of DMSP

The production of DMSP by algae is energetically costly [54], thus the regulation of DMSP in response to environmental change (e.g. hypersalinity) may be relatively slow [55]. However, the principal functions of DMSP in algae under varying environmental conditions are not well understood. Overall, a clear diel pattern in macroalgal intracellular DMSP and water column DMSPt concentrations was observed, with DMSP concentrations typically highest at night, supporting the macroalgal grazing defence hypothesis [51], [52], [53]. Our results also suggest that DMSP concentrations were driven, at least in part, by the reefal carbonate chemistry. Thus, intracellular DMSP may play a role in maintaining macroalgal cellular function whilst under low carbonate saturation conditions, although other factors may also play a role (e.g. nutrients, grazing pressure). In temperate red coralline algae, DMSP may be up-regulated in response to acute changes in saturation state [16], thus it may be proposed that the algae sampled in this study also respond to decreases in saturation state by up-regulating intracellular DMSP concentrations. DMSPt will have consisted of benthic, detrital, land and phytoplankton material. DMSPt on the reef flat was well described by dissolved oxygen and pH (81% deviance explained); whilst DMSPt on the reef crest was not well described by any of the measured abiotic parameters (most parsimonious parameter: ΩCa, 27% deviance explained), suggesting that DMSPt on the crest was influenced by offshore waters more than DMSPt on the reef flat (different phytoplanktonic community for example).

DMSP and its breakdown products have been proposed as an ‘antioxidant cascade’ [30], able to ‘mop up’ reactive oxygen species produced by photosynthesis or elevated temperature. Water temperature was a significant factor contributing to observed intracellular DMSP concentrations in *Amphiroa* sp. This suggests that either DMSP was utilised during the day and replenished at night or there was a lag in the response of intracellular DMSP in *Amphiroa* sp. to ROS production (perhaps due to the energy outlay required) [54]. However, such a long lag in DMSP response (seven hours or more) may not be an effective mechanism against oxidative damage.

Dissolved oxygen levels remained well above the suggested level for hypoxic impact (∼30% saturation) [56] and the effect of reduced oxygen saturation on the intracellular DMSP concentrations of macroalgae is not known. However, dissolved oxygen did appear to influence the intracellular DMSP concentrations of *Padina* sp. and *Turbinaria* sp., suggesting that DMSP may be up-regulated during periods of low oxygen saturation.

### Benthic Producers of DMSP

Tropical corals have been described as one of the most important benthic sources of DMSP and DMS in the coastal zone [39]. Most data are from the Great Barrier Reef (GBR), Australia but the sources of water column DMSP and DMS are undefined. The GBR is also morphologically and hydrographically different to the fringing reef of this study. Results from this study suggest that other macro-primary producers may be more important sources of DMSPt than corals. Nearshore DMSPt concentrations were highest in areas dominated by seagrass beds and macroalgae (on the reef flat) rather than corals (reef crest and slope). DMSPt concentrations were also elevated at the offshore site, where the primary source is likely to have been phytoplankton. Importantly, DMSP in the water column is available to microorganisms for potential breakdown into DMS, which is then available for atmospheric flux. Given the shallow water depth of the Suleman Reef platform (∼0.8 m), the inshore reef areas may be important sources of atmospheric DMS in the region.

### Structural Tolerance to Variable Conditions

Calcification in *Amphiroa* sp. (high-magnesium calcite) was visibly reduced on the reef flat, suggesting that the algae were, relatively speaking, partially decalcified in the more variable conditions. This could not be quantitatively confirmed but changes to the calcite structure of red coralline algae in response to variable carbonate saturation has been observed in the temperate coralline alga *Lithothamnion glaciale* [16]. Grazing pressure may have also contributed to the observed differences in calcification. In contrast, the calcification of *Padina* sp. (aragonite) was not significantly different between the reef flat and reef crest, despite previous reports of decalcified *Padina* sp. in naturally low pH environments in Italy and Papua New Guinea [23].

### Environmental Implications and Conclusions

In coastal regions, the effect of OA will be superimposed on the naturally variable environment, resulting in new carbonate extremes [3]. Our study suggests that intracellular DMSP concentrations may be regulated in response to variability in reefal carbonate chemistry, helping to maintain cellular function during periods of lower carbonate saturation. Macroalgal intracellular DMSP regulation may also impact the function of macroalgae within the reefal ecosystem. Coralline algae are important settlement cues for invertebrate larvae, particularly corals, e.g. [57], [58], a function which may be reduced under high-CO_2_ conditions [59] and which may be driven by intracellular DMSP concentrations [60]. It is projected that other calcifying reef organisms such as urchins may be detrimentally affected by OA, e.g. [22], [61], [62], impacting the food web dynamics of the reef ecosystem and perhaps allowing a phase shift towards macroalgae dominance to occur [63]. A shift towards macroalgal dominance may subsequently impact DMSP biogeochemistry, affecting ecosystem function and perhaps resulting in higher water column DMSPt concentrations. This will increase the potential atmospheric emissions of DMS from the area, with subsequent impacts on local climate regulation [26], [27].

## Supporting Information

Figure S1 **Diel pattern in abiotic parameters of Suleman Reef, Egypt.** *In situ* A: dissolved oxygen (%), B: photosynthetically active radiation (PAR, µmol photons m^−2^ s^−1^), C: *p*CO_2_ (µatm), D: HCO_3_^−^ concentration (µmol kg^−1^), E: CO_3_^2−^ concentration (µmol kg^−1^), F: pH.
(TIF)
