# Supplementary figures and images for: Spatiotemporal Variability of Dimethylsulphoniopropionate on a Fringing Coral Reef: The Role of Reefal Carbonate Chemistry and Environmental Variability
Source: PLoS One. 2013 May 28;8(5):e64651. doi: 10.1371/journal.pone.0064651 (PMC3665749; doi:10.1371/journal.pone.0064651)

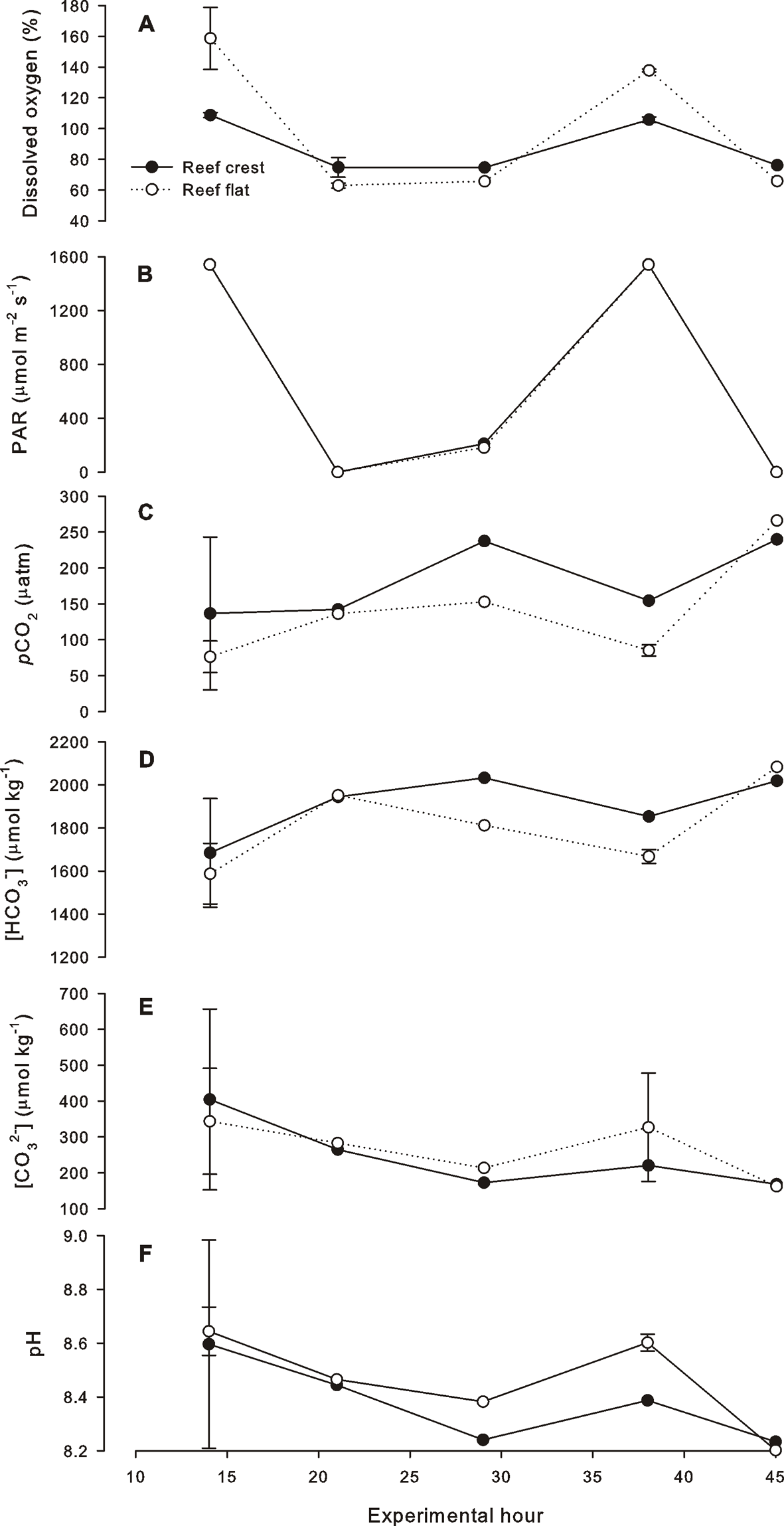

Supplement: Figure S1 — Diel pattern in abiotic parameters of Suleman Reef, Egypt. In situ A: dissolved oxygen (%), B: photosynthetically active radiation (PAR, µmol photons m−2 s−1), C: pCO2 (µatm), D: HCO3 − concentration (µmol kg−1), E: CO3 2− concentration (µmol kg−1), F: pH. (TIF) [file pone.0064651.s001.tif]
